# Evaluation of candidate reference genes stability for gene expression analysis by reverse transcription qPCR in *Clostridium perfringens*

**DOI:** 10.1038/s41598-022-23804-7

**Published:** 2022-11-13

**Authors:** Michele L. Williams, Mostafa Ghanem

**Affiliations:** 1 Department of Veterinary Medicine, University of Maryland, College Park, MD USA; 2 Virginia-Maryland College of Veterinary Medicine, College Park, MD USA

**Keywords:** Applied microbiology, Pathogens

## Abstract

Identification of stable reference genes for normalization purposes is necessary for obtaining reliable and accurate results of reverse transcription quantitative polymerase chain reaction (RT-qPCR) analyses. To our knowledge, no reference gene(s) have been validated for this purpose in *Clostridium perfringens*. In this study, the expression profile of ten candidate reference genes from three strains of *C. perfringens* were assessed for stability under various experimental conditions using geNorm in qbase + . These stability rankings were then compared to stability assessments evaluated by BestKeeper, NormFinder, delta Ct, and RefFinder algorithms. When comparing all the analyses; *gyrA*, *ftsZ*, and *recA* were identified within the most stable genes under the different experimental conditions and were further tested as a set of reference genes for normalization of alpha toxin gene expression over a 22-h period. Depending on the condition, *rpoA* and *rho* might also be suitable to include as part of the reference set. Although commonly used for the purpose of normalizing RT-qPCR data, the 16S rRNA gene (*rrs*) was found to be an unsuitable gene to be used as a reference. This work provides a framework for the selection of a suitable stable reference gene set for data normalization of *C. perfringens* gene expression.

## Introduction

*Clostridium perfringens* is a ubiquitous microorganism responsible for a wide array of diseases in animals and humans depending on its diverse toxin profile. It has the ability to produce highly resistant endospores that aid in transmitting disease as a result of their persistence in many different environments^1^. In addition to the production of alpha toxin which is present in all *C. perfringens* types, strains associated with various disease syndromes elaborate different toxins^2,3^. Based on these toxin profiles, *C. perfringens* is currently divided into seven toxinotypes- A through G^3^. The alpha toxin, or phospholipase C, of *C. perfringens* is encoded by the *plc* gene and is positively regulated by the two-component VirR/VirS system and the *agr* cell–cell signaling system^4^. Subtype B isolates cause hemorrhagic enteritis of sheep and produce beta toxin encoded by *cpb,* epsilon toxin encoded by *etx,* and often perfringolysin O (theta toxin) encoded by the *pfo* gene^2,5^. The Agr quorum sensing system regulates expression of *cpb* and *pfo*, but an as yet unidentified system regulates *etx* in these strains^5^. Isolates responsible for causing necrotic enteritis of poultry are usually associated with toxinotype G, which elaborate the NetB toxin^3,6^. This toxin is also regulated by the Agr-like quorum sensing system^7^.

One of the barriers to accurate and meaningful gene expression studies is the existence of validated reference genes for normalizing transcripts under specific experimental conditions. Normalization is a key step for producing reliable RT-qPCR assays because it controls for technically and experimentally induced variability in samples allowing for comparisons in target gene expression across these samples^8^. Historically, a single reference gene was chosen for normalization; however, the Minimum Information for Publication of Quantitative Real-Time PCR Experiments (MIQE) guideline indicates that this behavior is no longer acceptable unless proper evidence of constant expression under specific experimental conditions is provided^8^. As pointed out by Hellemans and Vandesomple^9^, performing pilot studies to assess stability of reference genes is an undertaking, and many researchers will rely on literature and colleagues to select reference genes even though the selected gene(s) may not be stable in the experimental system under consideration.

Reference genes for gene expression normalization have been validated for some clostridial species (*Clostridioides (Clostridium) difficle*^10^, *C. ljungdahlii*^11^ and *C. botulinum*^12^). Metcalf and colleagues^10^ established that *rrs*, *adk*, and *rpsJ* ranked as the most stable genes within the set of candidate genes in *C. difficile* when comparing expression during logarithmic growth and stationary growth. For *C. botulinum*, Kirk et al. established the stability of the 16S rRNA gene by determination of the coefficient of variation in Cq values over an 18-h time course; however, none of the commonly used analytic programs (geNorm^13^, normFinder^14^, BestKeeper^15^, refFinder^16^) were utilized to assess stability. For *C. ljungdahlii*, an important microorganism for biofuel production; *gyrA*, *fotI* (formate tetrahydrofolate ligase), and *rho* were recommended to be used as valid reference genes under the anaerobic growth with five different carbon sources as ranked by geNorm or NormFinder. The transcription level of 16S rRNA gene in this clostridial species, on the other hand, was found to be the least stable when assessed under these tested growth conditions^11^. There have been several studies using RT-qPCR to assess changes in relative gene expression in *C. perfringens* that used different reference genes for the normalization step^7,17–20^. In the Abildgaard study^17^, alpha toxin levels and expression were investigated using TaqMan analysis using *gyrA* (encoding DNA gyrase) or *rplL* (encoding the ribosomal protein L7/L12) as the reference genes for normalization. Both the Saito^18^ and Kawarizadeh^20^ studies utilized the 16S rRNA gene as the reference gene. In the Xiao study^19^, the genes *dnaE* and *sigH* were used to normalize expression values, whereas in the Yu^7^ study, *rpoA* was used for normalization. As the stability of the reference genes used in these studies was not documented, or single reference genes were used for normalization, it is necessary to establish reference gene stability prior to undertaking RT-qPCR analyses for relative gene expression quantification. In our current study, the expression profile and stability of 10 candidate reference genes were assessed in three different strains of *C. perfringens* subjected to three different experimental conditions (length of incubation, oxygen shock, and heat shock) in order to select a set of reference genes that could be used under a variety of experimental conditions. The expression of the *plc* gene, a virulence gene common to all strains of *C. perfringens*, at various time points during growth was then used to assess the reliability of our selected reference genes for use in normalization. To our knowledge, this is the first study in *C. perfringens* aimed to identify and validate reference genes for normalization of transcript levels for RT-qPCR analysis.

## Results

### Selection of the candidate reference genes and their amplification efficiencies

Ten genes were identified as potential reference genes from *Clostridium* species based on their previous use in this or other bacterial species. DNA sequences for eight candidate reference genes previously evaluated for *C. difficile* (*adk*, *gyrA*, *rho*, *rpsJ*, *tpiA*, *gluD* (*gdhA* in *C. perfringens*), *rpoA*, *rrs*)^10^ were acquired from NCBI for four *Clostridium perfringens* strains (ATCC 13,124, strain 13, EHE-NE18, and SM101) and aligned in MegAlignPro in order to identify highly (100%) conserved areas suitable for primer design. Two additional genes (*ftsZ* and *recA*) were chosen as they have been previously validated as suitable reference genes for other bacteria^21^. Species-specific primers for these targets were designed using Primer3^22,23^ (Table 1). Primers sets for *rpoA* and *rrs* were used as previously described as they had not been previously validated^7,24,25^.

**Table 1:** Candidate reference genes, functional groups, primer sequences, and expected amplicon sizes. Fragment sizes are based on alignment to *C. perfringens* ATCC 13124.

Gene	Functional category	Sequence (5’ – > 3’)	Size (bp)	Reference
*adk*	Nucleotide metabolism	F: TGGAAAATGTGATCTTTGCGGA	173	This study
		R: AGACCTCGTTTATTGCTTGTGT		
*gyrA*	DNA replication	F: GAGGTGGTAAGGGAATACAAGC	167	This study
		R: TGTTCCCTTAGCAGTTCTTCCT		
*recA*	DNA replication	F: CTGGTAAAACAACAGTGGCTTT	167	This study
		R: AGCTTGTTCTCCTGTATCTGGT		
*rho*	Transcription	F: TGAAAGACCAGAAGAAGTAACGG	150	This study
		R: ACATCTCTTCCTTGTTCAACCA		
*rpsJ*	Translation	F: ACAGGAGCAAAGGTTGTTGG	150	This study
		R: TGGTGATGGATTAACGATGTCT		
*tpiA*	Energy metabolism	F: GCATTCACTGGAGAAATCGCT	153	This study
		R: TGGAGTTAAGTTGTGTGCGA		
*ftsZ*	Cell division	F: AGGTAATTGGATGTGGAGGC	123	This study
		R: GGGCATGAGATAGTGTTAAGGC		
*gdhA*	Energy metabolism	F: GGTGGAGTAGCAACATCAGC	143	This study
		R: TGACCATATTCAGAAGCTGCA		
*rpoA*	Transcription	F: TTACCTGGAGTGGCTCCAAC	176	^7^
		R: ACACCTGGTCCTTGAGCATC		
*rrs*	Translation	F: GGGGGTTTCAACACCTCC	170	^24^ ^25^
		R: GCAAGGGATGTCAAGTGT		

Primer melting temperatures (Tm) are listed in Table 2 and corresponded to predicted Tms. Amplification specificity was confirmed for all primer pairs by visualizing single PCR amplicons of the correct size on agarose gel electrophoresis (Supplementary Fig. S1). Additionally, single peaks were observed for all 10 primer sets from dissociation curve analysis (Supplementary Fig. S2) indicating to absence of primer-dimers and non-specific amplification products.

**Table 2:** Features of the candidate reference genes.

Gene	Coefficient of determination (R^2^)	PCR efficiency [E (%)]- CFX maestro	PCR efficiency [E (SE)]- qbase +	Melting temperature (°C)
*adk*	0.997	1.82 (81.5)	1.81 (0.012)	76.0
*ftsZ*	0.996	1.83 (83.2)	1.82 (0.014)	76.5
*gdhA*	0.996	1.72 (72.4)	1.71 (0.010)	74.5
*gyrA*	0.997	1.73 (73.0)	1.73 (0.009)	76.5
*recA*	0.998	1.81 (81.0)	1.79 (0.013)	78.5
*rho*	0.995	1.83 (82.8)	1.81 (0.016)	75.0
*rpsJ*	0.997	1.86 (86.1)	1.83 (0.005)	77.5
*tpiA*	0.998	1.82 (82.4)	1.81 (0.004)	77.0
*rpoA*	0.997	1.75 (74.8)	1.74 (0.013)	76.0
*rrs*	0.997	1.78 (77.7)	1.76 (0.014)	84.0

Amplicons for each of the ten candidate reference genes were cloned into a cloning vector in order to generate standard curves for the calculation of amplification efficiencies. Amplification efficiencies for these genes ranged from 1.72 (*gdhA*) to 1.86 (*rpsJ*) with regression coefficients (R^2^) of 0.99 (Table 2).

## Establishment of experimental conditions

We chose to evaluate three varied growth conditions to simulate potential environments the bacterial cells could encounter during pathogenesis. The first condition (referred to as ‘incubation period’) assessed expression of the candidate reference genes after 4 h and 9 h of growth. These time points were chosen to approximate log phase and stationary phase growth; however, due to logistical constraints, real-time bacterial densities could not be monitored during the trial. For this trial, the density of bacterial growth equaled an OD_600_ of 0.15, 0.3, and 0.78 respectively for CP-1, CP-2, and CP-4 for the 4-h time point and an OD_600_ of 1.2, 1.3, and 1.4 respectively for CP-1, CP-2, and CP-4 for the 9-h time point.

In the second trial, two additional conditions were assessed. One condition (referred to as ‘oxygen shock’) evaluated expression of the candidate reference genes after a 30-min exposure to an aerobic environment following 4 h of growth under normal anaerobic conditions. No measurements of oxygen status were recorded. The other condition (referred to as ‘heat shock’) evaluated expression of the candidate reference genes after a 30-min exposure to 50 °C following 4 h of growth under normal 37 °C growth.

## Expression stability of candidate reference genes

The stability of transcription levels of the candidate reference genes under different stress conditions was initially evaluated by geNorm^13^ within the qbase + software package, version 3.2 (Biogazelle, Zwijnaarde, Belgium—www.qbaseplus.com). These results were then compared to analyses by additional algorithms─ BestKeeper^15^, comparative delta Ct^26^, normFinder^14^, and RefFinder^16^.

**Figure 1:**
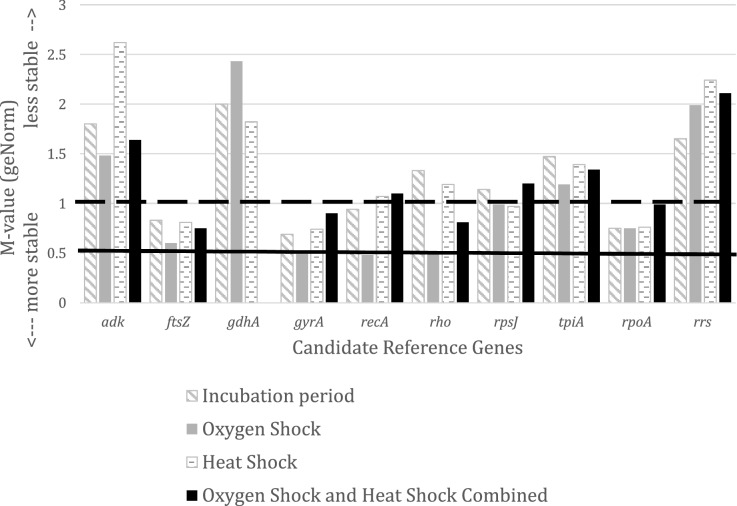
geNorm M-values for the 10 candidate reference genes for each set of growth parameters or stress condition using qbase + software. Medium stability (0.5 < average geNorm M-value < 1.0) is typically seen with heterogeneous samples.

A geNorm pilot experiment utilizing all 10 candidate reference genes produced M-values ranging from 0.69 (*gyrA*) to 2.0 (*gdhA*) when assessing stability only for the incubation period experimental condition (Fig. 1). Genes with medium stability, defined as having average geNorm M-values between 0.5 and 1.0, are often seen when heterogeneous sets of samples, such as our bacterial cultures, are evaluated^27^. Four genes fall into the category of medium stability when evaluating the incubation period condition (simulating log and stationary phase)—*ftsZ*, *gyrA*, *recA*, and *rpoA* or heat shock condition- *ftsZ*, *gyrA*, *rpoA*, and *rpsJ* (Fig. 1). Under our conditions, oxygen shock had the least influence on transcript levels of the candidate reference genes. Six out of the 10 candidate genes were considered stable after being subjected to oxygen shock with *ftsZ*, *gyrA*. *recA*, *rho*, *rpsJ*, and *rpoA* having M-values less than 1.0; *gyrA*, *recA*, and *rho* had M-values less than 0.5. Mean Cq values for an isolate and cycle variation, as indicated by standard deviation of the mean Cq values, across all conditions for a respective isolate are shown in Table 3. Average Cq values for any given gene would often differ by up to 10 Cq values between isolates for different growth/stress parameters indicating sample heterogeneity. The greatest variation is observed from all genes during comparisons during the incubation period trial (standard deviations range from 0.33 for CP-1 *rrs* to 6.47 for CP-2 *rpsJ*). The genes *gyrA* and *recA* showed the least variation in cycle number (standard deviation less than 1.0) for each isolate when assessing transcript levels during the oxygen shock and heat shock trials.

**Table 3:** Cycle variation across all samples for each growth/stress condition.

Gene	Isolate	Average Cq ± SD			
		Incubation period	Oxygen shock	Heat shock	All conditions
*adk*	CP-1	19.04 ± 4.82	18.22 ± 0.92	17.50 ± 1.08	18.22 ± 3.23
	CP-2	20.79 ± 4.74	25.42 ± 1.62	26.28 ± 2.47	24.42 ± 3.61
	CP-4	21.26 ± 3.10	19.31 ± 1.76	19.29 ± 1.11	19.81 ± 2.43
*ftsZ*	CP-1	18.93 ± 1.91	17.80 ± 1.04	18.31 ± 1.17	18.45 ± 1.48
	CP-2	19.32 ± 2.22	20.36 ± 0.40	21.02 ± 1.06	20.28 ± 1.75
	CP-4	20.88 ± 2.48	20.07 ± 0.59	20.46 ± 0.97	20.67 ± 1.69
*gdhA*	CP-1	23.11 ± 2.48	31.63 ± 2.25	34.69 ± 0	26.76 ± 4.84
	CP-2	21.77 ± 1.52	30.43 ± 1.23	33.81 ± 4.27	28.45 ± 6.27
	CP-4	24.72 ± 3.14	33.40 ± 3.81	33.78 ± 1.17	29.75 ± 5.60
*gyrA*	CP-1	17.94 ± 2.71	18.09 ± 0.74	18.01 ± 0.78	17.97 ± 1.79
	CP-2	16.98 ± 2.72	21.21 ± 0.77	21.36 ± 0.89	19.72 ± 2.89
	CP-4	19.28 ± 2.76	19.27 ± 0.17	19.54 ± 0.38	19.41 ± 1.77
*recA*	CP-1	18.21 ± 1.91	20.26 ± 0.45	19.43 ± 0.58	19.18 ± 1.55
	CP-2	17.63 ± 2.07	23.83 ± 0.58	23.45 ± 0.21	21.31 ± 3.30
	CP-4	18.91 ± 1.83	21.92 ± 0.30	21.34 ± 0.55	20.50 ± 1.79
*rho*	CP-1	19.64 ± 0.60	20.58 ± 0.67	21.15 ± 1.03	20.50 ± 1.05
	CP-2	20.20 ± 2.13	23.40 ± 0.86	23.96 ± 1.40	22.51 ± 2.48
	CP-4	20.83 ± 1.77	22.47 ± 0.45	23.57 ± 1.62	22.33 ± 1.97
*rpsJ*	CP-1	16.00 ± 4.04	16.29 ± 0.62	16.66 ± 0.70	16.29 ± 2.61
	CP-2	11.62 ± 6.47	22.24 ± 1.41	21.95 ± 1.12	20.05 ± 3.93
	CP-4	17.93 ± 2.84	18.50 ± 0.26	19.36 ± 1.09	18.66 ± 2.03
*tpiA*	CP-1	19.37 ± 2.08	19.18 ± 0.32	21.04 ± 2.12	20.05 ± 2.37
	CP-2	21.47 ± 3.48	26.08 ± 0.26	26.75 ± 0.89	24.55 ± 3.93
	CP-4	20.96 ± 1.64	22.91 ± 0.67	22.99 ± 0.82	22.29 ± 1.60
*rpoA*	CP-1	17.24 ± 2.82	15.08 ± 1.06	15.84 ± 0.81	16.12 ± 2.14
	CP-2	15.24 ± 4.71	19.07 ± 1.47	19.32 ± 1.72	18.99 ± 2.34
	CP-4	19.20 ± 3.26	17.01 ± 0.36	17.41 ± 0.46	18.00 ± 2.31
*rrs*	CP-1	4.66 ± 0.33	5.47 ± 3.10	6.70 ± 3.00	5.30 ± 2.47
	CP-2	6.79 ± 1.74	4.15 ± 1.27	7.70 ± 4.48	6.80 ± 3.22
	CP-4	6.16 ± 1.09	7.17 ± 3.45	7.86 ± 4.25	7.48 ± 2.93

To compare the stability ranking determined by qbase + software to other algorithms, Cq data was submitted to the online tool RefFinder (as found at www.heartcure.com.au/reffinder/). RefFinder^16^ is a comprehensive tool that integrates the output of other computational algorithms by assigning weight to each candidate genes then calculating a final ranking based on the geometric mean of the weighting value for each gene. In this process, the tool provides the output for each of the additional computational programs. Comparisons of the expression stability ranking calculated by RefFinder for geNorm, BestKeeper, NormFinder, and the delta Ct method, as well as the comprehensive RefFinder ranking are shown in Table 4. For most of the algorithms and conditions, the genes *ftsZ*, *recA*, and *gyrA* were consistently within the top four ranks. The BestKeeper^15^ computational algorithm showed the most different ranking of the 10 genes under the incubation period condition compared to the other analysis platforms with *rrs*, *rho*, and *gdhA* identified as the three most stable genes. Two of these genes (*rrs* and *gdhA*) were identified among the least stable genes by the other platforms. RefFinder comprehensive rankings identified *ftsZ*, *recA*, and *gyrA* consistently within the top four ranks when comparing any of the conditions (incubation period, oxygen shock or heat shock, all conditions together) with geometric means of rank values between 1.41 and 3.71 depending on the growth/stress condition being evaluated. Under two conditions (incubation period and all conditions together), RefFinder identified *rho* as the third most stable gene with a geometric mean of the rank value at 3.36 and 2.51, respectively. Under these same conditions, *rpoA* was not determined to be within the top four most stable genes (rank 9 for incubation period and rank 5 for all conditions together).

**Table 4:** Expression stability ranking of the ten candidate reference genes according to geNorm, BestKeeper, NormFinder, Delta CT, and the RefFinder comprehensive analysis. These analyses were performed using the web-based RefFinder tool found at www.heartcure.com.au/reffinder/.

Ranking	**GeNorm**						**BestKeeper**						
	Incubation period		Oxygen shock and heat shock		All conditions		Incubation period		Oxygen shock and heat shock		All conditions		
	Gene	M value	Gene	M value	Gene	M value	Gene	ST Dev	Gene	ST Dev	Gene		ST Dev
1 (Most stable/ best)	***ftsZ ***	0.73	***gyrA ***	0.894	***ftsZ ***	1.215	*rrs*	1.33	***recA***	1.135	***ftsZ***		1.621
1	***recA***	0.73	***recA***	0.894	***gyrA***	1.215	*rho*	1.49	***gyrA***	1.205	***gyrA***		1.805
3	***gyrA***	0.853	***ftsZ***	1.153	***rpoA***	1.515	*gdhA*	1.86	***ftsZ***	1.228	***recA***		1.936
4	*rho*	1.054	***rpoA***	1.317	***recA***	1.768	***recA***	1.93	***rpoA***	1.561	*rho*		2.11
5	*tpiA*	1.298	*rho*	1.683	*rho*	1.92	***ftsZ***	2.2	*rho*	2.022	*rrs*		2.321
6	*rpsJ*	1.527	*rpsJ*	1.953	*rpsJ*	2.144	*tpiA*	2.64	*rpsJ*	2.388	***rpoA***		2.322
7	***rpoA***	1.662	*adk*	2.258	*adk*	2.348	***gyrA***	2.7	*rrs*	2.762	*rpsJ*		3.061
8	*rrs*	1.818	*rrs*	2.682	*rrs*	2.625	*rpsJ*	3.21	*adk*	3.405	*adk*		3.386
9	*adk*	1.954	*gdhA*	3.463	*tpiA*	3.392	***rpoA***	3.48	*gdhA*	4.322	*tpiA*		4.305
10 (Least stable)	*gdhA*	2.121	*tpiA*	4.257	*gdhA*	3.944	*adk*	3.62	*tpiA*	5.36	*gdhA*		5.677

Ranking	**Delta CT**						**Normfinder**						
	Incubation period		Oxygen shock and heat shock		All conditions		Incubation period		Oxygen shock and heat shock		All conditions		
	Gene	Avg STDEV	Gene	Avg STDEV	Gene	Avg STDEV	Gene	Stability value	Gene	Stability value	Gene		
1 (Most stable/ best)	***ftsZ***	1.56	***rpoA***	3.07	***gyrA***	2.93	***ftsZ***	0.365	***rpoA***	0.782	*rho*		0.303
2	***recA***	1.64	***gyrA***	3.19	*rho*	3.08	***recA***	0.411	*rho*	0.984	***gyrA***		1.158
3	***gyrA***	1.65	***recA***	3.26	***recA***	3.15	***gyrA***	0.53	***gyrA***	1.523	***recA***		1.537
4	*rho*	1.84	*rho*	3.35	***ftsZ***	3.2	*rho*	1.086	***ftsZ***	1.599	***ftsZ***		1.666
5	*tpiA*	2.06	***ftsZ***	3.37	***rpoA***	3.23	*tpiA*	1.395	***recA***	1.811	***rpoA***		1.729
6	*rpsJ*	2.28	*rpsJ*	3.95	*rpsJ*	3.71	*rpsJ*	1.829	*adk*	2.684	*adk*		2.464
7	***rpoA***	2.38	*adk*	4.09	*adk*	3.73	***rpoA***	1.937	*rpsJ*	3.085	*rpsJ*		2.699
8	*rrs*	2.39	*rrs*	4.84	*rrs*	4.2	*rrs*	1.996	*rrs*	3.923	*rrs*		3.209
9	*adk*	2.62	*gdhA*	6.03	*tpiA*	6.07	*adk*	2.257	*gdhA*	5.215	*tpiA*		5.498
10 (Least stable)	*gdhA*	2.79	*tpiA*	7.44	*gdhA*	6.15	*gdhA*	2.477	*tpiA*	7.038	*gdhA*		5.573

Ranking	**RefFinder comprehensive ranking**												
	Incubation period		Oxygen shock and heat shock		All conditions								
	Gene	Geomean of ranking values	Gene	Geomean of ranking values	Gene	Geomean of ranking values							
1 (Most stable/ best)	***ftsZ***	1.50	***gyrA***	1.86	***gyrA***	1.41							
2	***recA***	2.00	***recA***	1.97	***ftsZ***	2.00							
3	*rho*	3.36	***rpoA***	2.00	*rho*	2.51							
4	***gyrA***	3.71	***ftsZ***	3.66	***recA***	3.22							
5	*rrs*	4.76	*rho*	3.76	***rpoA***	4.61							
6	*tpiA*	5.52	*rpsJ*	6.24	*rpsJ*	6.48							
7	*rpsJ*	6.45	*adk*	6.69	*adk*	6.96							
8	*gdhA*	7.40	*rrs*	7.74	*rrs*	7.11							
9	***rpoA***	7.45	*gdhA*	9.00	*tpiA*	9.00							
10 (Least stable)	*adk*	9.24	*tpiA*	10.00	*gdhA*	10.00							

The four genes identified by geNorm in the qbase + software as most stable are indicated in boldface

## Expression profile over time of the alpha toxin gene (plc)

**Figure 2:**
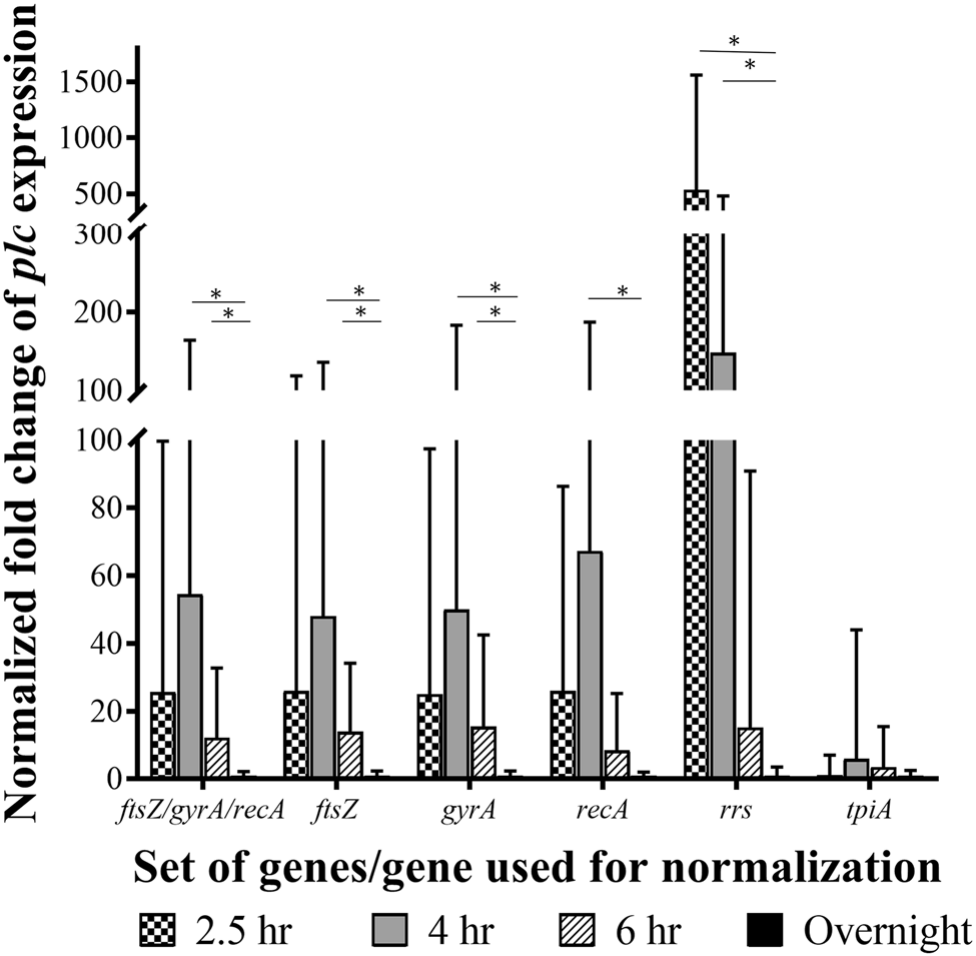
Relative expression of alpha toxin (*plc*) during growth of *Clostridium perfringens* over a 22-h period. The expression data were normalized using *ftsZ*, *gyrA*, *recA*, *rrs*, or *tpiA* as reference genes individually and with the geometric mean of *ftsZ* + *gyrA* + *recA.* 22-h samples were considered the control for calculations. *P *≤ 0.05 shown by *

It has long been recognized that alpha toxin production occurs during active cellular growth^17,28–30^, so evaluating the expression of *plc* at various time points during growth should allow us to evaluate the reliability of our selected reference genes for use in normalization of transcript levels. The incubation time points we evaluated were 2.5 h and 4 h for simulating early- and mid-log phase, and 6 h and 22 h for simulating early- and late-stationary phase. Because of the variability in growth rates between our three *C. perfringens* isolates, it was difficult to achieve the desired growth phase targets (early-log, mid-log, early-stationary) for all three strains when cells were harvested at our experimental time points. The actual OD_600_ of the cultures used for RNA harvest are shown in Supplementary Table S1. Expression of the *plc* gene relative to expression of this gene after 22 h of growth was assessed following normalization with our previously determined set of reference genes (*ftsZ*, *recA*, and *gyrA*), with these genes individually, with one of the least stable but reliably amplified genes (*tpiA*), and with *rrs* as a historically used reference gene (Fig. 2). Our set of reference genes and the same genes individually as reference genes produced similar fold changes in *plc* expression; however, using the set of reference genes more often produced more consistent results indicated by shorter error bars. When a Student’s t-test was used to assess statistical significance (*p *≤ 0.05) of the changes in expression at 4 h in relation to the 22-h time point, a maximal and significant upregulation of *plc* transcription was observed when employing *ftsZ*, *gyrA*, and *recA* either as a set or individually. Although the transcript levels decreased by 6 h, the level of *plc* expression was still significantly upregulated when normalizing with the reference set genes or *ftsZ* and *gyrA* individually, but not *recA* individually.

When *tpiA*, one of the less stably expressed candidate reference genes, was used for normalization; the same pattern of expression was observed but to a greatly depressed level. Expression of *plc* at the 4-h time point was maximal with 9 times less transcript observed when *tpiA* was used for normalization as compared to the same expression when normalization was performed using the set of three reference genes (~ sixfold increase vs 54-fold increase, respectively). None of the change in *plc* expression were found to be significant when *tpiA* was used in the normalization process.

When *plc* expression was normalized with *rrs*, the apparent relative expression of *plc* decreased over time instead of increasing during active growth. As such, normalization with the 16S RNA gene produced a different expression profile for *plc* than was observed when using our reference set genes (either as a set or individually). Significant upregulation of *plc* at the 2.5-h time point was only noted when *rrs* was utilized for normalization.

## Discussion

*Clostridium perfringens* causes a wide variety of diseases in humans and animals as a result of the toxins it produces^2^. These toxins are regulated by a complex network of signaling pathways and understanding how different environmental conditions modulate the expression of these toxins will provide us with a better understanding of the role of these toxins the development of disease. Reverse transcription qPCR is the gold standard method in studying expression of specific genes and virulence regulation under different environmental conditions^31^, and proper normalization is essential to achieve reliable results^8^. Under the most appropriate normalization strategy, selected reference genes should be verified to be stably expressed under the physiological state or experimental conditions under investigation^8^. To identify suitable genes for normalization of gene expression in *C. perfringens*, we performed the necessary validation analyses under three different conditions that could be relevant during pathogenesis.

In this study, we selected and evaluated ten candidate reference genes (*adk*, *ftsZ*, *gyrA*, *gdhA*, *recA*, *rho*, *rpoA*, *rpsJ*, *rrs*, and *tpiA*) for use in normalization of transcript levels in *C. perfringens*. As suggested by others, we selected genes representing different functional classes to minimize the likelihood of co-regulation^10,32^ (Table 1). Of these genes; *rrs*, *gyrA*, *recA*, *rpoA*, *rho*, *ftsZ*, and *adk* have all been experimentally tested for use in other bacteria; however, the stability for each gene was not validated in all studies^21^. For instance, *adk* has been experimentally tested in 3 different organisms (*Bacillus cereus* group strains^33^, *C. difficile*^10^, and *C. botulinum*^12^), yet it was only identified as stably expressed in *C. difficile*. Three of the 10 genes investigated (*gyrA*, *rrs,* and *rpoA*) have been used previously as an endogenous control for *C. perfringens,* although validity for these genes was not documented^7,17,18,20,34^.

In our study, we determined the most suitable reference genes for normalization in *C. perfringens* to be *ftsZ*, *gyrA*, and *recA*. This determination was made by assessing the rankings of genes by all the available computational algorithms under the different growth/stress conditions and choosing the genes that ranked highest (within the top four) for the different conditions by each of the computational algorithms. The three genes of our proposed reference set ranked within the top spots under all conditions (analyzed separately and together) by geNorm, NormFinder, and RefFinder (Table 4). These three genes also ranked highest using the delta Ct analysis method when assessing stability under the incubation period trial and when results of all the experimental conditions were combined; whereas *gyrA* and *recA* ranked second and third, but *ftsZ* ranked fifth when assessing expression in the oxygen shock and heat shock experiment. The delta Ct and geNorm methods are similar in that a stepwise comparison of expression values is made to exclude or rank less favorably genes that fluctuate expression under different conditions. Other genes, especially *rho* and *rpoA*, occasionally were ranked in the top 4, but this ranking was not consistent between conditions and computational algorithms.

The 16S rRNA gene (*rrs*) is frequently used as a reference gene for normalization; yet it was not found to be stably expressed in this study. One reason for this instability is likely linked to the considerable variation in *rrs* transcript levels where they were often extremely abundant (Cq values < 10) and difficult to reproduce consistently as the high abundance makes it difficult to accurately set the baseline^13^. Another reason the *rrs* gene is not appropriate as a reference gene is that *rrs* transcript levels were quite abundant in relation to the transcript levels of the other genes including the *plc* gene as our gene of interest (Cq less than 10 vs. Cq values around 20), and its use is not appropriate as its abundance is overrepresented in relation to the total amount of mRNA in the samples^8^. In fact, when we used *rrs* as the internal control for normalization of *plc,* a decreasing upregulation of the alpha toxin gene over time was observed, which is not the same expression profile observed when using the genes in our proposed reference set (Fig. 2).

One hurdle of this study, but one that is likely common for researchers studying bacterial populations with a high degree of heterogeneity, is the inherent variability in gene expression between different strains of the same bacterial species. In *C. difficile*, a discrepancy between reference gene stability attributable to the heterogeneity of *C. difficile* ribotypes was observed^10^. In this study, we used three *C. perfringens* isolates representing three different toxinotypes─ types B, E, and G. Each isolate showed different growth kinetics with CP-2 (type E) growing most rapidly and CP-1 (type B) growing slowest. Similarly, each isolate differed in the expression levels at the different sampling points. To illustrate this variability in Cq values, we can look at *recA* and *tpiA* expression across all the conditions between the different strains. In the oxidative stress experiment, for *recA* transcripts, there was approximately a 3 Cq value spread between the three strains, but close values between the conditions within a strain as evidenced by the low standard deviations (average Cq ± SD values for CP-1, CP-2 and CP-4 are 20.26 ± 0.45, 23.83 ± 0.58, and 21.92 ± 0.30, respectively; Table 3). On the other hand, for *tpiA* in the same experiment, there was approximately a 6 Cq value spread between the three strains, but close Cq values between the conditions within a strain as evidenced by the low standard deviations (average Cq ± SD values for CP-1, CP-2 and CP-4 are 19.18 ± 0.32, 26.08 ± 0.26, and 22.91 ± 0.67, respectively; Table 3).

For robust qPCR assays, the general rule-of-thumb is to have near 100% amplification efficiency as this indicates that the amount of the target doubles at each cycle^8^. The range for a good PCR is 90 – 110% with a range of 20% (E = 1.9–2.1). A potential pitfall of this study is our amplification efficiencies are not within this range; however, they are close to each other. Our PCR efficiencies ranged from 72 to 86% with the efficiencies of our chosen reference set between 73% (*gyrA*) to 83.2% (*ftsZ*). This range is narrower than the range of efficiency for a “good” qPCR (10 vs. 20%). So although these efficiencies are lower than we would like, they are similar enough to result in accurate relative quantification^35^.

To test the ability of the proposed set of *C. perfringens* reference genes to be used for normalization, we applied *ftsZ*, *gyrA*, and *recA* transcript levels for normalization of the alpha toxin gene (*plc*) at different stages of growth. Ohtani and colleagues^4^ noted that toxin gene expression in *C. perfringens* is unique as it is initiated much earlier during growth thereby reaching maximum expression during the exponential phase of growth rather than stationary phase of growth. Ohtani and colleagues^4^, by using Northern blot analysis, attributed this expression pattern to the *agrBD* system in *C. perfringens* rather than a traditional LuxS-based quorum-sensing mechanism. Using RT-qPCR, Abildgaard and colleagues^17^ observed a similar induction of *plc* during early exponential phase with a subsequent decrease in expression occurring by stationary phase. This is the same expression profile observed in this study when using our proposed reference set for normalization. We found that using our proposed reference genes, either in combination or individually, produced similar expression trends over the tested time points from early exponential phase (2.5-h) to late stationary phase (22-h). The observance of the transcript level peaking supports previous findings that mRNA expression is induced rather than constitutive^17,29^.

Selection of stable reference genes for data normalization is required for reliable and accurate RT-qPCR analysis. In this study, we assessed potential reference gene stability of three different *C. perfringens* strains representing three different toxinotypes under three different experimental conditions that could have physiologic relevance. Overall, we recommend the use of *gyrA*, *ftsZ*, and *recA* as a set of reference genes for use in *C. perfringens* for normalization of gene transcripts in relative expression studies. While we know that ideal universal endogenous control genes have not been identified and likely do not exist^13^ especially for species like *C. perfringens* that are heterogeneous nature, the proposed set of reference genes identified in this study will provide researchers with a good starting point for developing an accurate normalization strategy for their *C. perfringens*-based expression assay. Ideally, these reference genes should be confirmed as stable under other experimental conditions or with additional strains prior to their use.

### Methods

## Strains, media, and growth conditions

Two strains of *C. perfringens* were purchased from ATCC belonging to toxintype B (ATCC 3626, CP-1) and toxintype E (ATCC 27,324, CP-2). A third strain containing *netB* (C103-100, CP-4) was obtained from Auburn University (Ken Macklin, extension specialist and professor) that originated from the Mitchem-Sparks Regional Diagnostic Laboratory in Boaz, Alabama. This *netB*-containing isolate is classified as subtype G in the recently expanded toxin-based typing scheme^3^.

Bacterial cultures were grown on perfringens agar (HiMedia, Kennett Square, PA) for routine maintenance and in BHI (Criterion, Hardy Diagnostics, Santa Maria, CA) broth during experimental assays. An anaerobic environment was maintained in standard sized growth chambers containing gas-generating sachets with anaerobic indicators (Becton, Dickinson and Company; Franklin Lakes, NJ). Starter BHI cultures were inoculated from plates and allowed to grow anaerobically overnight (~ 16 h) at 37 °C. Trials were carried out as two experiments. In Experiment 1 for the incubation period trial, 10 ml BHI cultures were inoculated using 200 µl of the appropriate starter culture, then allowed to grow 4 h to approximate the logarithmic phase (target OD_600_ 0.3–0.4) or 9 h to approximate the stationary phase (target OD_600_ 1.2–1.4). After the appropriate time points, the cultures were divided into 2 ml aliquots, centrifuged at 3,260 X g for 4 min, then pellets were stored at − 80 °C. For experiment 2, the oxygen shock and heat shock trials, all cultures were inoculated and incubated as for the incubation period samples in Experiment 1. At 4-h, the control samples were divided into 1.5 ml aliquots, centrifuged at 14,000 × g for 2 min, then stored at − 80 °C or immediately processed for RNA extraction. The oxygen shock samples were removed from the anaerobic growth chamber and incubated aerobically at 37 °C for 25 min, after which time 1.5 ml aliquots were centrifuged at 14,000 × g for 2 min, then stored at − 80 °C or immediately processed for RNA extraction. For the heat shock samples, cultures were transferred to a 50 °C water bath for 30 minutes^36^, after which time 1.5 ml aliquots were centrifuged at 14,000 × g for 2 min, then stored at − 80 °C or immediately processed for RNA extraction.

## Primers

Ten genes were identified as potential reference genes from the pertinent *Clostridium* sp. literature (Table 1). Eight *C. perfringens*-specific primers were designed for the genes utilized by Metcalf *et al*^10^ for *C. difficile*. Primers were designed using Primer3web^22,23^ with the following parameters: optimal primer size-20 bp (range 18 bp–23 bp); Tm- 59 °C (range 57–62 °C), GC content- 50% (range 30–70%). Using Primer-BLAST against the non-redundant (nr) nucleotide database, potential primers were assessed to check that *C. perfringens* entries were the only match at > 95% similarity for the primer set. Primers sets for *rpoA* and *rrs* were used as previously described^7,24,25^. All primers were synthesized by Integrated DNA Technologies (IDT, Coralville, IA) and purified by standard desalting methods.

## Determination of PCR efficiency

All 10 putative reference genes were amplified, purified (GeneJet PCR Purification Kit, Life Technologies, Carlsbad, CA), and cloned into pMiniT 2.0 cloning vector (NEB PCR Cloning Kit, New England Biolabs, Ipswitch, MA) to establish a standard curve for the determination of the PCR efficiency. Clones were transformed into *E. coli* (NEB 10-beta competent *E. coli*, New England Biolabs), and colonies were screened by PCR. Bacterial cells containing plasmids with proper inserts were expanded in LB amended with 100 mg/ml carbenicillin to facilitate extraction of the plasmids (Monarch Plasmid Miniprep kit, New England Biolabs). Inserts were verified by sequencing. Plasmid DNA concentrations were measured spectrophotometrically using a Nanodrop 1000 (Thermo Scientific, Wilmington, DE). A 6-point tenfold dilution series (concentration ranges from 10 ng/µl to 100 fg/µl) was prepared. Ten µl reactions were prepared in quadruplicate for each reference gene-containing plasmid using PowerUp SYBR Green Master Mix (Applied Biosystems) and 500 nM of each primer in the respective primer set. Amplification was performed using a CFX-96 Connect real time thermal cycler (Bio-Rad, Hercules, CA) with the following cycling parameters: 50 °C UDG activation for 2 min, 95 °C DNA polymerase unlock step for 2 min, followed by 40 cycles of 95 °C for 15 s, 61 °C for 15 s, 72 °C for 1 min with fluorescent images captured at the completion of each cycle. The amplification was immediately followed by a dissociation curve analysis to check for primer-dimer formation and any nonspecific amplification products.

## RNA isolation and reverse transcription

Total RNA was isolated from the stored pellets of *C. perfringens* isolates harvested at logarithmic and stationary phases of growth using the Promega SV Total RNA kit (Promega Corporation, Madison, WI) following the manufacturer’s protocol for Isolation of RNA from Gram-Positive and Gram-Negative Bacteria with a few modifications. Pellets were resuspended in 100 μl of freshly prepared TE containing 20 mg/ml lysozyme and incubated at room temperature for 10 min. Purified RNA was eluted with 100 μl nuclease-free water. A post-elution DNase treatment was performed (TURBO DNA-free kit, Invitrogen). RNA concentrations were assessed spectrophotometrically using a Nanodrop 1000 (Thermo Scientific, Wilmington, DE) prior to normalization of RNA concentration. Concentration of normalized RNA was subsequently verified by Nanodrop to be within 10% of each other.

## Reference gene validation

Ten µl reactions were prepared in duplicate for each *C. perfringens* cDNA with each of 10 potential reference genes using PowerUp SYBR Green Master Mix (Applied Biosystems) and 500 nM of each primer of the respective primer set. No reverse transcriptase controls were included for each RNA isolation. Amplification was performed in clear low profile plates using a CFX-96 Connect real time thermal cycler (Bio-Rad, Hercules, CA) with the following cycling parameters: 50 °C UDG activation for 2 min, 95 °C DNA polymerase unlock step for 2 min, followed by 40 cycles of 95 °C for 15 s, 61 °C for 15 s, 72 °C for 1 min with fluorescent images captured at the completion of each cycle. Amplification was immediately followed by dissociation curve analyses to check for primer-dimer formation and any nonspecific amplification products.

Cq values for each candidate reference gene were analysed for stability using geNorm^13^ within the qbase + software package, version 3.2 (Biogazelle, Zwijnaarde, Belgium—www.qbaseplus.com). Comparisons of stability rankings were made following additional computational analyses using BestKeeper^15^, comparative delta Ct^26^, normFinder^14^, and RefFinder^16^. Cq values for all candidate reference genes from experiments of this study are included in supplementary Table S2.

## Alpha toxin gene (plc) qPCR

The set of reference genes identified by geNorm to be most stable under these stress conditions were used to normalize expression of *plc* in response to growth phase. Primers were designed using the same parameters as for the candidate reference genes to amplify a 174 bp fragment of the *plc* gene of *C. perfringens* (forward primer 5′- CTG ACA CAG GGG AAT CAC AA- 3′, reverse primer 5′- CAT GTC CTG CGC TAT CAA CG- 3′). As alpha toxin has been shown to be regulated by cell density mechanisms, the growth time points we assessed were 2.5 h (early-log), 4 h (mid-log), 6 h (early stationary), and 22-h (late stationary). Bacteria were grown and harvested at each experimental time point as previously described. Procedures for RNA isolation, cDNA synthesis, and RT-qPCR conditions were the same as for the reference gene validation experiments. Stability of the reference genes under these growth conditions were confirmed with geNorm (qbase +). Normalized gene expression of *plc* was determined using the multi-gene normalization quantification model^27^ and statistical significance of *plc* expression due to growth phase in relation to the 22-h time point simulating stationary phase when alpha toxin gene expression should be minimal was assessed using a two-tailed Student’s t-test (*p *≤ 0.05).

## Supplementary Information

Supplementary Information 1.

Supplementary Information 2.

## Data Availability

All data relevant to the study are included in the article or uploaded as supplementary information. In addition, the datasets used and/or analyzed during the current study are available from the corresponding author on reasonable request.
