# Supplementary material for: Evaluation of candidate reference genes stability for gene expression analysis by reverse transcription qPCR in Clostridium perfringens
Source: Sci Rep. 2022 Nov 13;12:19434. doi: 10.1038/s41598-022-23804-7 (PMC9659559; doi:10.1038/s41598-022-23804-7)
Supplement: Supplementary file 1 — Supplementary Information 1. [file 41598_2022_23804_MOESM1_ESM.pdf]

Supplementary Data

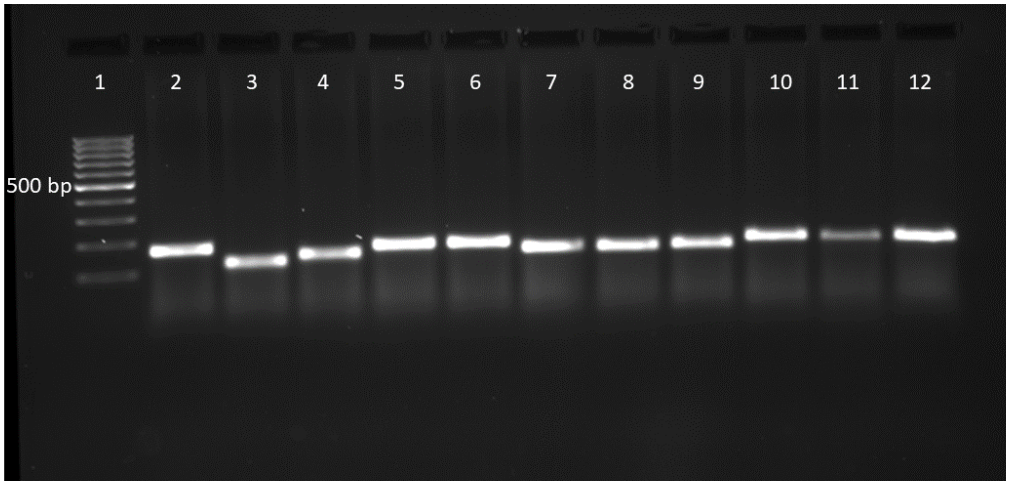

Figure S1.Agarose gel (2%) electrophoresis showing single amplification products for each primer set confirming the specificity of the primers for each gene. Lane 1- GeneRuler 100 bp DNA ladder (ThermoScientific), 2- *adk*, 3- *ftsZ*, 4- *gdhA*, 5- *gyrA*, 6- *recA*, 7- *rho*, 8- *rpsJ*, 9- *tpiA*, 10- *rpoA*, 11- *rrs*, 12- *plc*.

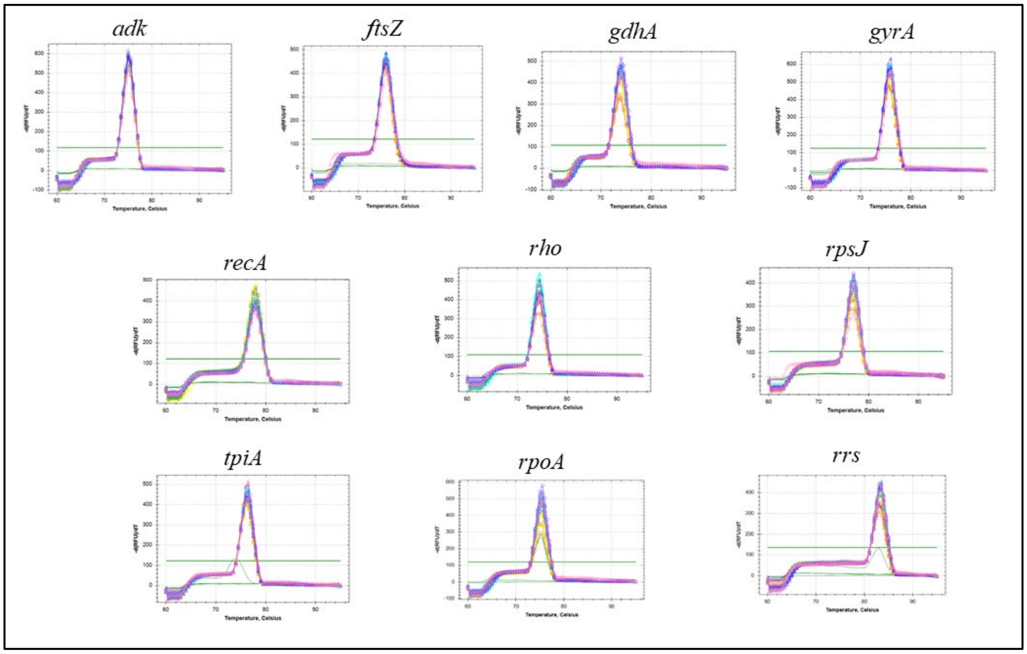

Figure S2. Dissociation-curve analysis showing single peaks indicating the absence of primer-dimers and nonspecific amplification products.

Table S1. Variation in growth rates of *C. perfringens* for harvesting RNA. Optical density (OD) measurements were not recorded for the 22-hour overnight time point. Time 0 readings are provided for evaluation purposes only; cells for this time point were not harvested for RNA.

Sample	OD_{600} (nm)			
	Time 0	Time 2.5 hr	Time 4 hr	Time 6 hr
CP-1	0.044	0.256	0.811	0.816
CP-2	0.076	0.659	1.297	1.283
CP-4	0.074	0.457	1.157	1.262
